# Surgical Anatomical Structure of Uterine Artery Caudal Space for Robot-Assisted Extrafascial Hysterectomy With Ureterohypogastric Nerve Fascia Preservation: A Case Description

**DOI:** 10.7759/cureus.54512

**Published:** 2024-02-20

**Authors:** Koji Shimabukuro, Maiko Ichikawa, Takafumi Tsukada, Seiichi Endo, Masae Sakamoto

**Affiliations:** 1 Department of Obstetrics and Gynecology, Tsuchiura Kyodo General Hospital, Tsuchiura, JPN

**Keywords:** vesicohyogastric fascia, ureterohypogastric nerve fascia, robot-assisted surgery, extrafascial hysterectomy, uterine artery caudal space

## Abstract

A surgical technique has been developed using a robot-assisted system to create the surgical anatomical structure of the uterine artery caudal space (UACS), a landmark for performing extrafascial hysterectomy, enabling a safe and easy parametrial resection to prevent ureteral injury and cervical sidewall bleeding at hysterectomy. UACS is created to preserve the ureterohypogastric nerve fascia (UHNF), which envelopes the ureter and the hypogastric nerve, and the vesicohypogastric fascia (VF), which wraps the uterine artery and veins. The boundaries of UACS are UHNF laterally, the uterine cervix medially, and VF cranially. VF is penetrated between UACS and the medial pararectal space under the uterine vessels and transected. We present a case of early-stage uterine cancer and describe the new surgical technique in detail, using UACS as a surgical landmark. This surgical technique could be applied not only to early-stage uterine cancer but also to benign uterine tumors using a robot-assisted surgical system.

## Introduction

Ureteral injury at hysterectomy for benign uterine tumors is reported to occur at rates of 0.03-1.5% [1-3], and the most common site of ureteral injury is the distal ureter [1,4]. Two options usually chosen for benign uterine tumors to prevent ureteral injury are subtotal hysterectomy, which leaves the cervix intact, and intrafascial hysterectomy, which involves an incision in the lateral wall of the cervix. In intrafascial hysterectomy, bleeding from the incised cervix is an issue. This is especially true in laparoscopic surgery, where achieving hemostasis is challenging and thermal damage to the urinary tract due to the use of energy devices is a concern [5].

In extrafascial hysterectomy, a surgical operation does not affect the cervical fascia and avoids bleeding from the uterine side by not incising it; however, extrafascial hysterectomy requires the technique of ureteral tunnel dissection in order to expose the distal ureter. This is a step of the procedure that might cause ureteral injury during a surgical operation.

Ureterohypogastric nerve fascia (UHNF) is a layer of connective tissue that wraps the ureter, mesoureter, and hypogastric nerve and continues to the fascia surrounding the urinary bladder [6,7]. Vesicohypogastric fascia (VF) is a layer of connective tissue that envelopes the internal iliac vessels and their branches to pelvic organs, including the uterine artery and veins and continues to the cervical fascia [6,8]. Preservation of UHNF and VF keeps the ureter and uterine vessels intact at hysterectomy, results in a bloodless operation, and prevents ureteral injury.

We have invented a new surgical technique that utilizes the benefits of robot-assisted surgery. It involves the creation of a surgical anatomical structure, the uterine artery caudal space (UACS), between UHNF and the uterine cervix. The new surgical technique enables safe and easy extrafascial hysterectomy without dissecting the ureteral tunnel, making it applicable to benign uterine tumors as well as malignant ones using a robot-assisted surgical operation system.

## Case presentation

**Figure 1:**
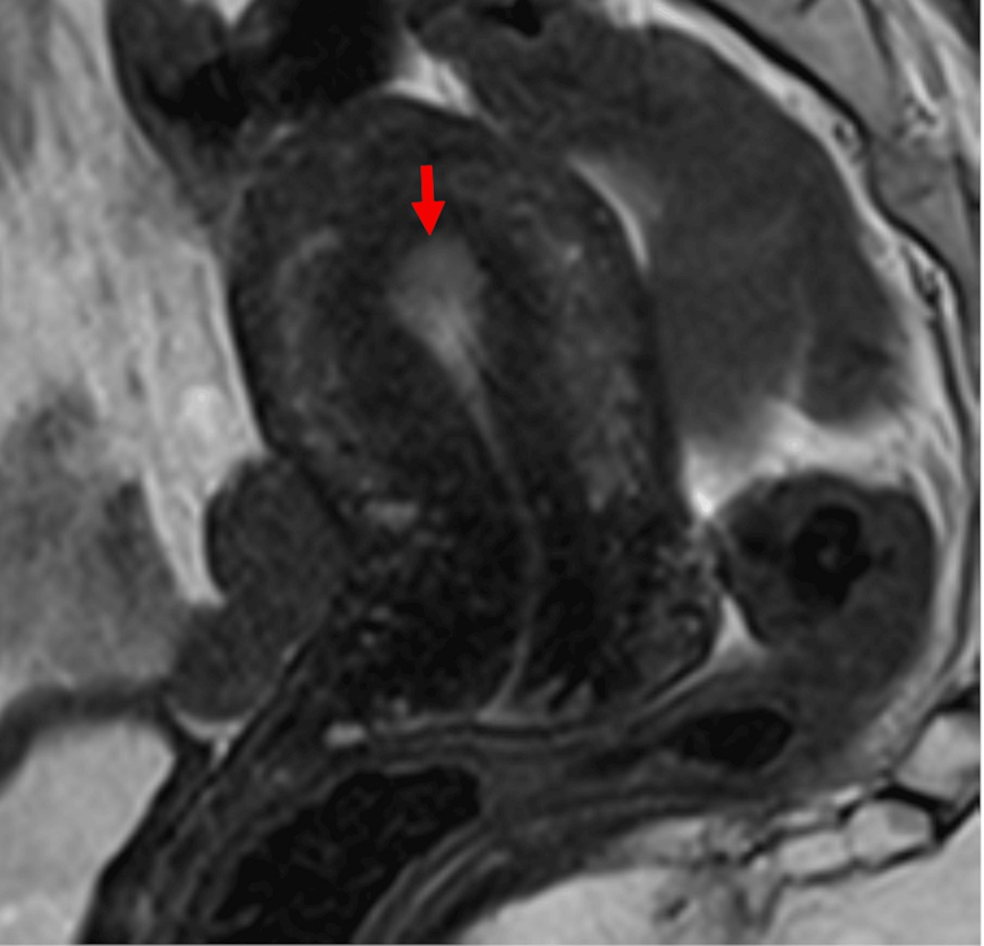
Midsagittal section of the uterus T2-weighted imaging on MRI shows an 8 x 13 mm endometrial tumor (red arrow) without myometrial invasion and indicates stage IA endometrial cancer. MRI: magnetic resonance imaging

A 50-year-old woman, gravida 3, para 2, presented to our hospital with a complaint of abnormal uterine bleeding. She had open surgical histories of appendectomy, myomectomy, and ovarian cystectomy for endometriosis. She was diagnosed with Grade 1 endometrioid carcinoma by an endometrial biopsy. The MRI examination revealed an 8 x 13 mm endometrial tumor with no myometrial involvement (Figure 1), and a negative result of metastases on CT scans indicated a presumptive diagnosis of stage IA endometrial cancer in the International Federation of Gynecology and Obstetrics staging system of endometrial cancer in 2023 [9], clinical stage T1aN0M0. She was scheduled for a robot-assisted total hysterectomy with a bilateral salpingo-oophorectomy.

Intraoperative findings

**Figure 2:**
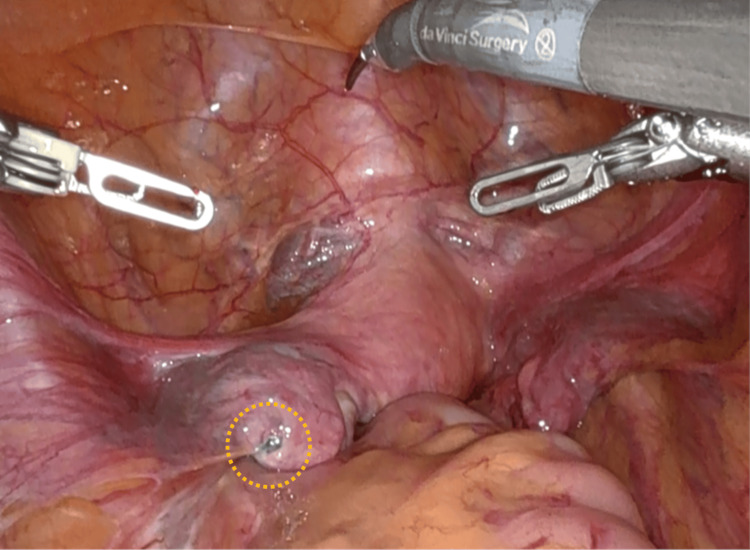
Intraoperative findings An endometriotic blueberry spot is detected on the left adnexa (yellow dotted circle).

The uterus was nearly normal in size, and the right adnexa was free of any gross lesions. An endometriotic blueberry spot was detected on the left adnexa, and the left fallopian tube was occluded (Figure 2).

Robot-assisted surgical technique

**Figure 3:**
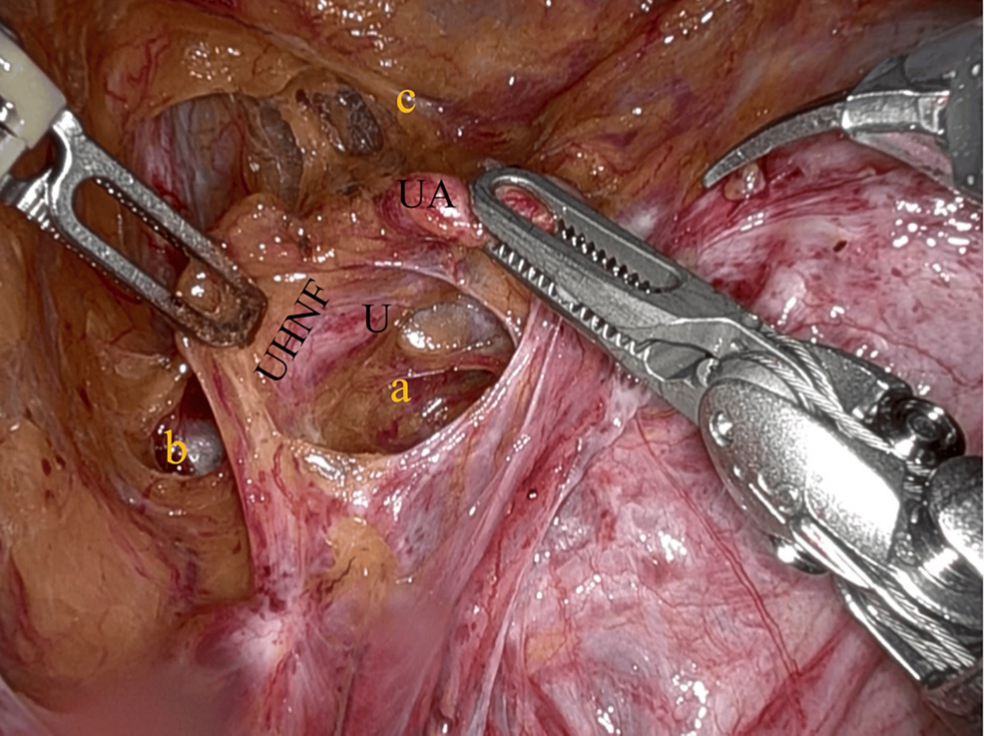
Creation of pararectal spaces and dissection of the UHNF The UA is retracted upward, and the medial pararectal space (Okabayashi) (a) is developed as the UHNF is dissected. The lateral pararectal space (Latzko) (b), the paravesical space (c), and U. UA: uterine artery; UHNF: ureterohypogastric nerve fascia; U: ureter

The key surgical steps of the robot-assisted extrafascial hysterectomy are described as follows: after the vesicouterine peritoneum and the anterior leaf of the broad ligament were incised, the bladder was mobilized to the anterior vaginal fornix, and the bladder pillars were dissected. The uterine artery was exposed and retracted upward by the fourth arm (Cadiere forceps) of da Vinci X (Intuitive Surgical, Inc., California, USA), and then UHNF was dissected by the first arm (fenestrated bipolar forceps) and the second arm (monopolar shears) to open the lateral and medial pararectal space (Latzko and Okabayashi space) (Figure 3) [8,10-11].

**Figure 4:**
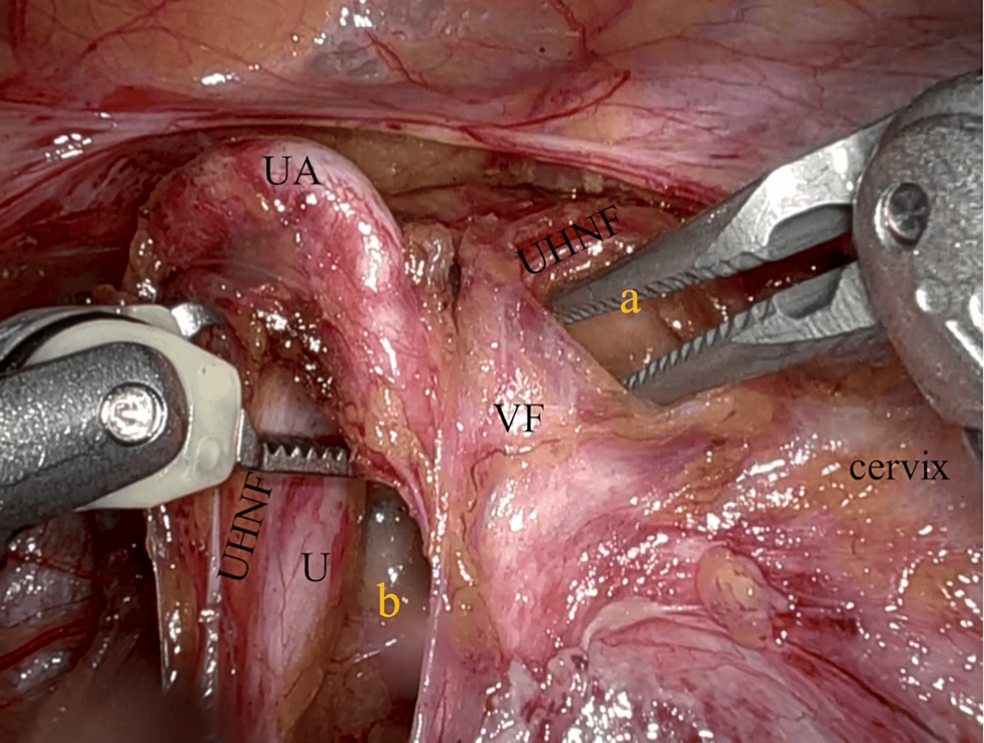
Creation of UACS The UACS (a) is being developed bluntly as the VF, which envelopes the UA and veins, is retracted upward. VF extends into the cervical fascia; therefore, the preservation of VF leads to a bloodless operation. The UHNF is shown to be conserved as the lateral boundary of UACS (a) and the medial pararectal space (b), U. UACS: uterine artery caudal space; VF: vesicohypogastric fascia; UA: uterine artery; UHNF: ureterohypogastric nerve fascia; U: ureter

**Figure 5:**
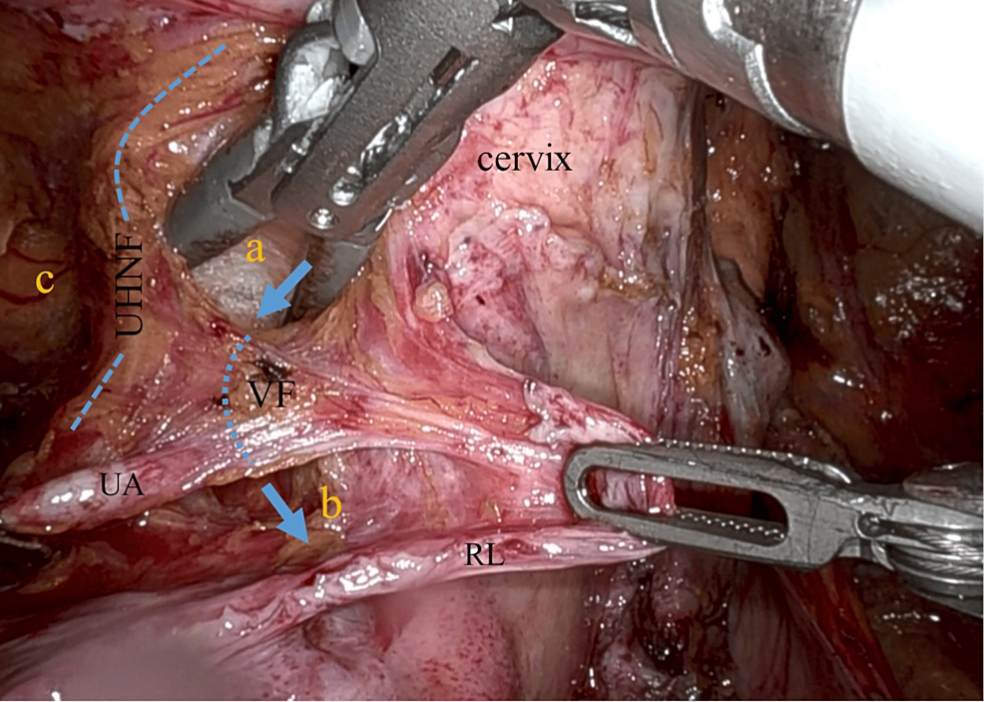
Dissection of UHNF and penetration of VF The VF medial to the UHNF is being penetrated under the uterine vessels from UACS (a) to the medial pararectal space (Okabayashi) (b) (blue arrows and dotted line). The paravesical space (c), the RL, UA, and the ridgeline of UHNF (blue dashed line). VF: vesicohypogastric fascia; UHNF: ureterohypogastric nerve fascia; UACS: uterine artery caudal space; RL: round ligament; UA: uterine artery

**Figure 6:**
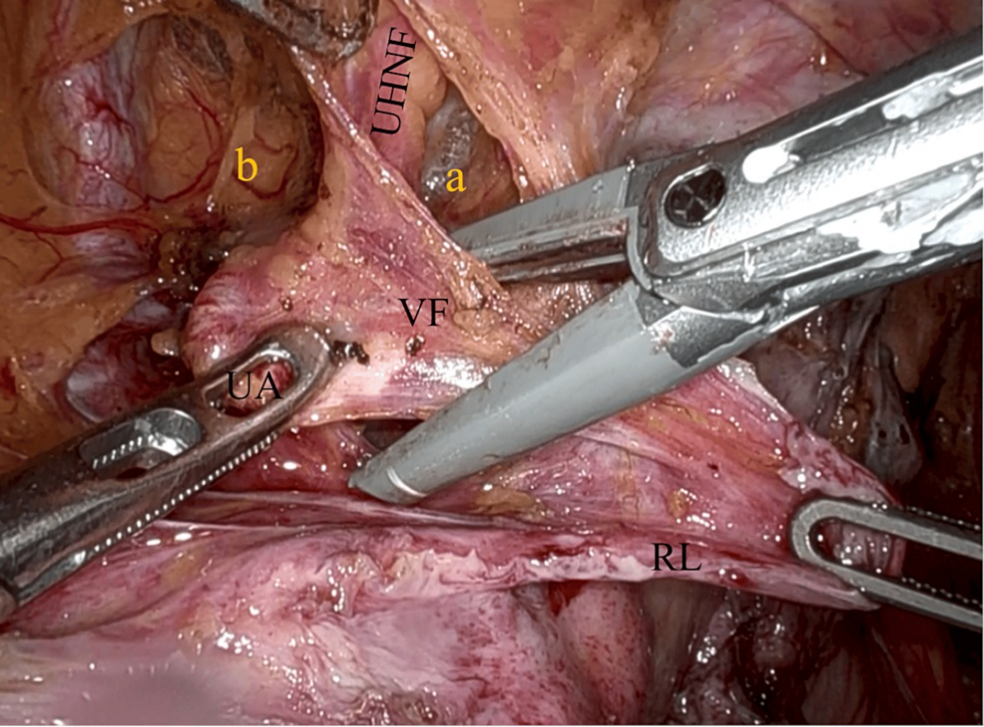
First resection of parametrium after dissection of UHNF The VF, which envelopes the UA and veins, is being clamped. The UHNF is dissected between UACS (a) and the paravesical space (b), the RL. VF: vesicohypogastric fascia; UA: uterine artery; UHNF: ureterohypogastric nerve fascia; UACS: uterine artery caudal space; RL: round ligament

The uterine artery was then retracted cranially, and UACS was created sharply and bluntly at the paracervix (Figure 4). UACS is bordered by UHNF on the lateral side, the uterine cervix on the medial side, and VF (parametrium) on the cranial side, enveloping the uterine artery and veins (Figure 4) [6,8]. VF was penetrated under the uterine vessels (Figure 5), which were then sealed and cut by the second arm (the vessel sealer extended) (Figure 6).

**Figure 7:**
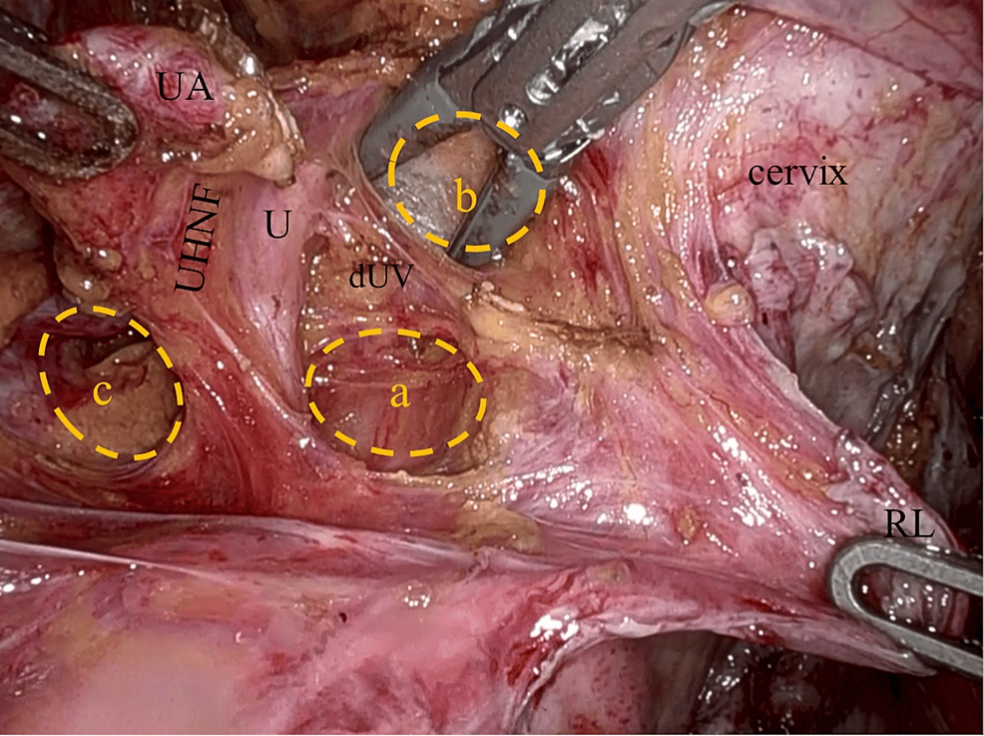
Second resection of parametrium after dissection of UHNF The dUV and the uterine branch of the hypogastric nerve are clearly shown before the second parametrial resection. The U is enveloped by the UHNF courses along the lateral boundary of the medial pararectal space (Okabayashi) (a) and UACS (b). No uterine sidewall bleeding is seen after the first resection of the parametrium. The lateral pararectal space (Latzko) (c), UA, U, and RL. UHNF: ureterohypogastric nerve fascia; dUV: deep uterine veins; UACS: uterine artery caudal space; UA: uterine artery; U: ureter; RL: round ligament

**Figure 8:**
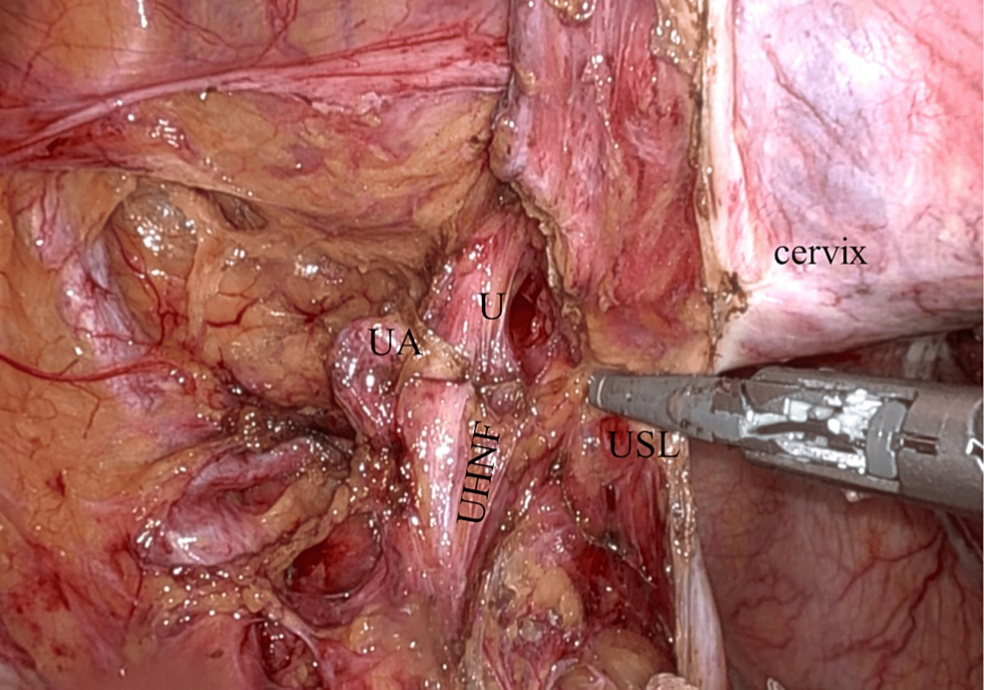
Preservation of UHNF and resection of USL The USL is coagulated and cut after the procedure of extrafascial parametrial resection. The medial side of the UHNF is seen. UHNF: ureterohypogastric nerve fascia; USL: uterosacral ligament; UA: uterine artery; U: ureter

**Figure 9:**
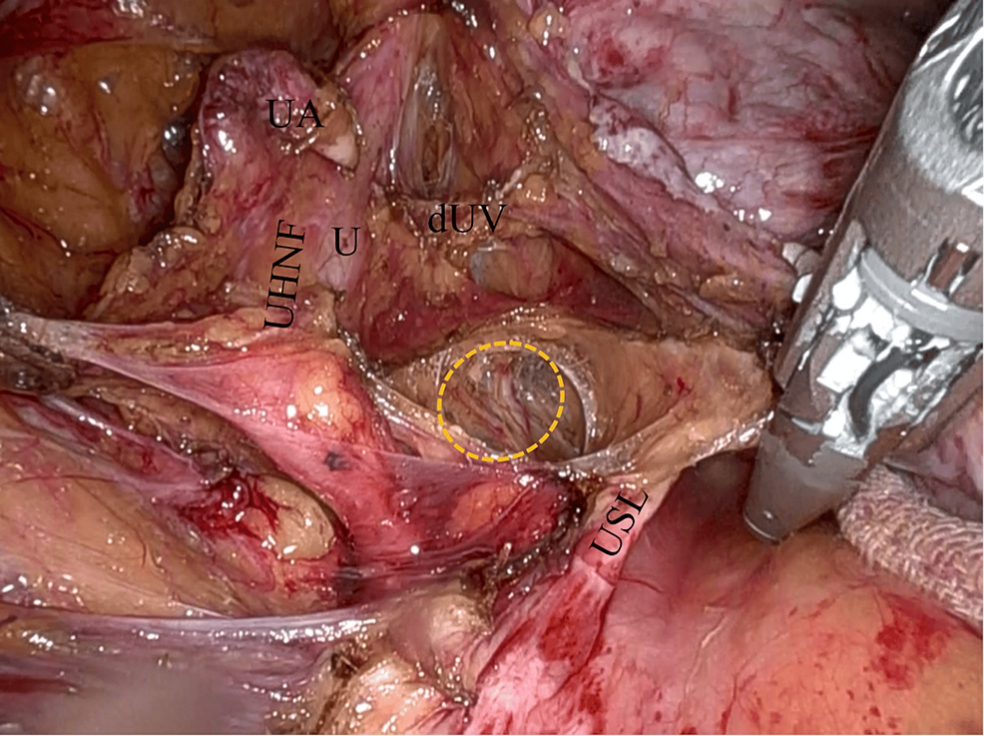
Hypogastric nerve in UHNF The medial pararectal space is widely exposed, and the UHNF is inadvertently partly peeled to show the hypogastric nerve (yellow dashed circle) directly. The USL, the dUV, the UA, and the U. UHNF: ureterohypogastric nerve fascia; USL: uterosacral ligament; dUV: deep uterine veins; UA: uterine artery; U: ureter

The deep uterine veins and the uterine branch of the hypogastric nerve in the remaining parametrial tissue were sealed and cut (Figure 7), and then the uterosacral ligament was coagulated and cut (Figure 8). In this case, UHNF was inadvertently partly peeled to show the hypogastric nerve directly (Figure 9).

The same steps were repeated on the other side, and the subsequent surgical procedures were performed in the usual manner. The console time was 144 minutes with an estimated blood loss of 5 ml.

The post-operative course was uneventful, except for a skin rash caused by a disinfectant. Pathology of the surgical specimen showed a 17 x 10 mm exophytic tumor of Grade 1 endometrioid carcinoma confined to the endometrium with no lymphovascular space involvement in the uterus and endometriosis in bilateral ovaries.

## Discussion

For benign uterine tumors, intrafascial hysterectomy is often performed to avoid ureteral injury. However, a disadvantage of this procedure is the persistent bleeding from the uterine sidewall. As a result, particularly in laparoscopic hysterectomy, there is a tendency to apply coagulation energy devices multiple times, which raises concerns about delayed ureteral injury due to thermal damage.

Extrafascial hysterectomy could prevent bleeding from the cervical sidewall. However, to minimize the risk of inadvertent clamping, suturing, or coagulation on the paracervical tissue leading to ureteral injuries, meticulous dissection of the ureteral tunnel is essential. The distal ureter should be exposed from the uterine artery crossing point to its terminal part. Despite these precautions, there remains a risk of ureteral injuries associated with the dissection procedure. Therefore, extrafascial hysterectomy is typically performed for early-stage uterine cancer or suspected malignancies.

In addition, the ureteral tunnel dissection procedure is seldom carried out by general gynecologists other than gynecological oncologists who perform radical hysterectomy for cervical cancer. Yin et al. reported that the incidence of ureteral injury in open and laparoscopic radical hysterectomy in early-stage cervical adenocarcinoma is 0.4% and 2.4%, respectively [12], indicating that the ureteral tunnel dissection procedure in laparoscopy is a difficult technique even for gynecologic oncologists. The proposed extrafascial hysterectomy technique creates extra space between UHNF and the uterine sidewall in the region caudal to the uterine artery, allowing for lateral displacement of the ureter. This technique enables the safe resection of VF (which corresponds to the parametrium) without the need for ureteral tunnel dissection.

VF envelopes the internal iliac vessels, the umbilical artery anteriorly, and their medial branches to the bladder, the uterus, and the rectum as well; accordingly, the fascia of the cardinal ligament of the uterus and the lateral ligament of the bladder and the rectum is the continuation of VF; furthermore, VF extends its continuation to the fascia of the respective organs [6-8]. VF, which envelopes uterine vessels, crosses UHNF to reach the uterine cervix; the uterine artery crisscrosses UHNF over the ureter; and the uterine veins pierce UHNF under the ureter [6,8,11]. VF medial to UHNF, which corresponds to parametrium, is clearly shown by the development of UACS and the medial pararectal space (Okabayashi), perforated between the two spaces under the uterine veins, and then coagulated and resected. In this case, the second resection of the parametrium, which contains the deep uterine veins with the uterine branch of the hypogastric nerve (cardinal ligament), was also needed.

The tissue in the paracervical region is so densely interwoven that it is difficult to develop UACS in open surgery or even in laparoscopic surgery; however, the space creation is much easier with the magnified view and more precise operation of robot-assisted surgery. As our new technique skips the procedure of uterine tunnel dissection with the risk of ureteral injury, it could be allowed in benign uterine tumors with robot-assisted surgery. So far, this procedure has been successfully performed on other cases of early uterine cancer and benign uterine tumors. Further studies by other surgeons are needed to show this procedure as a safe alternative for the management of benign uterine tumors as well.

## Conclusions

Occasionally, intrafascial hysterectomy results in uterine side bleeding, whereas extrafascial hysterectomy may lead to ureteral injuries. In order to avoid both risks, we have devised the above-mentioned new surgical technique to ensure the safe operation of extrafascial hysterectomy. The crucial point of this technique is the extra space that can be created in the caudal vicinity of the uterine artery at the paracervix by using a surgical robot with its signature advantage of magnified view and precise movement. This space creation leads to the lateral displacement of the distal ureter wrapped in UHNF. The new technique allows for a safe and complete extrafascial hysterectomy without requiring the complex skill of dissecting the distal ureter. Therefore, it would be very useful, especially to general gynecologists who mainly deal with benign tumors. For substantiation of its usefulness, follow-up studies are needed to prove that the reported procedure is a superior alternative for the removal of benign uterine tumors as well as early-stage malignant uterine tumors.
